# Is readiness for interprofessional learning maintained until graduation? A longitudinal study of medical students

**DOI:** 10.5116/ijme.6aab.f620

**Published:** 2026-09-25

**Authors:** Takami Maeno, Ryohei Goto, Junji Haruta, Ayumi Takayashiki, Sachiko Ozone, Masatsune Suzuki, Tetsuhiro Maeno

**Affiliations:** 1 Center for Medical Education, The Jikei University School of Medicine; 2 Institute of Medicine, University of Tsukuba, Tsukuba, Japan; 3 Medical Education Center, School of Medicine, Keio University, Tokyo, Japan

**Keywords:** Interprofessional education, undergraduate medical education, medical student, longitudinal study, community-based medical education

## Abstract

**Objectives:**

This study aimed to examine changes in medical students' readiness for interprofessional learning before and after community-based medical education (CBME), and whether these changes are sustained until graduation.

**Methods:**

This longitudinal observational study included medical students at T University who participated in CBME during the 2018–2019 and 2019–2020 academic years. Data were collected as part of routine curriculum evaluation, with written informed consent obtained for research use. Participants were followed prospectively until graduation, with earlier data obtained from interprofessional education (IPE) programs. Readiness for interprofessional learning was assessed using the Japanese version of the Readiness for Interprofessional Learning Scale (RIPLS). The primary analysis focused on changes before CBME (T5), after CBME (T6), and at graduation (T7). A one-way repeated-measures analysis of variance (ANOVA) with Greenhouse–Geisser correction was conducted, followed by Bonferroni-adjusted post hoc comparisons.

**Results:**

Analyses were conducted among participants with complete data at T5–T7 (n = 192). RIPLS scores increased after CBME, peaked at T6, and decreased at graduation. Repeated-measures ANOVA showed a significant effect of time (F_(1.60, 305.28) _= 31.50, p < .001, ηp² = 0.14). Post hoc analyses showed higher scores at T6 than T5 (p < .001) and lower scores at T7 than T6 (p < .001), with no difference between T5 and T7 (p = .062).

**Conclusions:**

Readiness for interprofessional learning increased after CBME but was not sustained until graduation. Strengthening continuity between undergraduate IPE and postgraduate training may help sustain readiness and support transition to clinical practice.

## Introduction

To provide high-quality care in response to increasingly complex medical needs, healthcare professionals must acquire the competency to collaborate across professional boundaries. Interprofessional education (IPE) has become a critical foundation for developing this competency.^1^ IPE is promoted as an integral part of undergraduate education, and there has been a rapid increase in the number of universities integrating IPE into their curricula.^2^ As future physicians who will be part of interprofessional healthcare teams, medical students are expected to develop positive attitudes toward interprofessional collaboration during undergraduate education. Studies show that medical students exposed to IPE before graduation tend to adopt a more positive attitude toward interprofessional work.^3^^,^^4^ Therefore, IPE plays a crucial role in preparing medical students for collaborative practice.

Evidence supporting the educational effectiveness of undergraduate IPE is increasing. A systematic review^5^ found that about half of the study results showed a significant improvement in positive attitudes toward IPE when introduced during pre-clinical and clinical years following the intervention. Guraya's review^6^ highlighted improvements in students' knowledge, skills, and attitudes when comparing pre- and post-program evaluations. However, many studies that analyze the effects of IPE rely on pre-post or post-only designs, which limit understanding of how these attitudes change throughout the entire medical curriculum.

The spiral curriculum model, which introduces IPE early and progressively builds on it according to learners' levels, is recommended to optimize its effectiveness.^2^ The spiral curriculum model emphasizes repeated exposure to important learning themes across different stages of education.^7^ This progressive sequencing is particularly relevant to IPE, as readiness for collaboration is expected to develop cumulatively through repeated learning experiences across educational stages. However, longitudinal studies examining changes in readiness for interprofessional learning have reported inconsistent findings. For example, previous studies have reported both declines in readiness over time and improvements following IPE interventions.^8^^,^^9^ In addition, the number of such longitudinal studies remains limited.^10^

Early classroom-based IPE during pre-clinical years can be reinforced by later clinical experiences, such as community-based medical education (CBME), where IPE is embedded in real clinical settings. CBME provides an important opportunity for students to observe and participate in interprofessional collaboration and has been reported to enhance learning related to collaborative practice.^11^ Given that the goal of undergraduate IPE is to prepare students for collaborative work in interprofessional teams upon graduation, it is crucial to understand whether improvements in readiness are sustained during the final stage of undergraduate medical education. However, it remains unclear whether changes in readiness for interprofessional learning following CBME are maintained during this final stage.

The Readiness for Interprofessional Learning Scale (RIPLS) is commonly used to assess students’ attitudes toward collaborative practice. This scale was developed to evaluate readiness for interprofessional learning, as changes in attitudes are considered essential for effective interprofessional learning among students participating in IPE.^12^ RIPLS assesses learners’ readiness to acquire the knowledge, skills, and behaviors required for collaboration within interprofessional teams.

While many studies have demonstrated the immediate impact of IPE programs on students’ attitudes, evidence regarding the long-term sustainability of these effects remains limited. This gap is particularly important at the final stage of undergraduate medical education, which represents the transition to collaborative clinical practice.  Therefore, this study aimed to examine changes in medical students' readiness for interprofessional learning before and after CBME and whether these changes are sustained until graduation, while also providing a descriptive overview of longitudinal changes across the medical curriculum.

## Methods

### Study design: Longitudinal observational study

#### Participants

The study included medical students at T University from two academic years who participated in CBME, conducted mainly during the fifth year and extending into the early sixth year, between October 2018 and May 2019 (CBME 2018–2019 group) and between October 2019 and May 2020 (CBME 2019–2020 group).

This study was designed as a longitudinal observational study. At the start of CBME, students were informed about prospective follow-up until graduation and the use of previously collected data from earlier IPE programs conducted during their second and third years, and written informed consent was obtained.

For the CBME 2018–2019 group, the follow-up survey at graduation was conducted in January 2020. Data collected during the second-year IPE program in 2015 and the third-year IPE program in 2016 were used. For the CBME 2019–2020 group, the follow-up survey at graduation was conducted in January 2021. Data collected during the second-year IPE program in 2016 and the third-year IPE program in 2017 were used.

This study was approved by the Ethics Committee of the University of Tsukuba. The questionnaire was conducted as part of the curriculum evaluation, and written informed consent was obtained from all participants for the use of their data for research purposes.

#### Curriculum of medical students in Japan

The standard duration of education at medical schools is six years. Typically, students enroll in medical schools after graduating from high school and spend the first few years studying general liberal arts courses. Concurrently, medical students undertake basic medicine courses for one to two years, followed by clinical medicine, which includes internal medicine and surgery, for an additional one to two years. Before beginning clinical training (clinical clerkship), medical students must pass a nationwide computer-based testing (CBT) and a pre-clinical clerkship objective structured clinical examination (OSCE) to earn the title of "student doctor." Subsequently, they engage in clinical training at university hospitals, regional hospitals, or clinics for 1 to 1.5 years during their fourth or fifth year of medical school. Following clinical training, students are required to pass the post-clinical clerkship OSCE and the university graduation exam to qualify for graduation. They must then pass the National Medical Practitioners Qualifying Examination, which is held annually. This examination consists of 400 multiple-choice questions administered over two days and does not include a practical skills test. The pass rate for the examination is approximately 90%.^13^

#### Overview of the IPE Program at T University

The IPE program at T University's School of Medicine is structured to allow students to learn in stages, beginning with early exposure immediately after they enter the university and progressing to CBME, which forms a part of the clinical training for fifth and sixth-year students.

#### Second-year IPE program

The four-hour second-year IPE program is mandatory. Participants include second-year medical students from T University and second-year nursing, physical therapy, occupational therapy, and radiological technology students from I University. The program employs team-based learning as its instructional method. In a large classroom setting, students are organized into small, mixed groups of six to seven members, where they discuss patient care using case scenarios.

#### Third-year IPE program

The third-year IPE program is a mandatory, one-week-long course that includes third-year medical students, fourth-year nursing students, third-year clinical laboratory technician students from T University, and fifth-year pharmaceutical students from R University. The program employs a problem-based learning approach, where students are organized into small, mixed groups of eight to nine members. In these groups, they discuss strategies for working together to solve the problems of patients and their families.

#### CBME for fifth- and sixth-year students

After completing a year of clinical training in internal medicine and surgery at university hospitals, starting in October of their fourth year, medical students undertake a compulsory practical training period of two to three weeks at a community clinic or small hospital from October of the fifth year to May of the sixth year. The timing of this practical training period varies among students. With the goal of learning about interprofessional collaboration, medical students participate in home nursing or home rehabilitation and accompany staff from various professions during their work.

#### Measurement

We used the Japanese version of the Readiness for Interprofessional Learning Scale (RIPLS)^12^^,^^14^ to measure the educational effect of IPE. This scale, developed as a self-administered questionnaire, assesses health-professional students' readiness for interprofessional learning. Tamura and colleagues evaluated the validity and reliability of the Japanese version of the RIPLS.^14^ This version includes 19 items divided into three subscales: "teamwork and collaborations" with 13 items, "IPE opportunities" with two items, and "uniqueness of profession" with four items. The items are rated on a five-point Likert scale, ranging from "strongly agree" to "strongly disagree." Responses were scored from 5 to 1, with higher scores indicating stronger agreement, and five items were reverse-scored. The total RIPLS score was calculated as the sum of the 19 items (range: 19–95). The RIPLS score reflects the readiness of professionals for IPE. Consequently, higher RIPLS total scores indicate a greater readiness of undergraduate healthcare students for interprofessional learning. In this study, the RIPLS total score was used for analysis, as previous studies have reported concerns regarding the reliability of its subscales.^14^

The measurement time points were defined as follows: T1 and T2 were before and after the second-year classroom-based IPE program, T3 and T4 were before and after the third-year classroom-based IPE program, T5 and T6 were before and after CBME, and T7 was at graduation. The timeline of educational stages and measurement time points is shown in Figure 1. The internal consistency of the RIPLS total score was acceptable across all measurement time points (Cronbach’s α = 0.87–0.92).

Age and sex were collected at T3. Age at T5 was estimated by adding two years to the age recorded at T3, based on the standard progression of the curriculum.

The medical education department at T University managed the responses to the questionnaire. After merging the data from T1 to T7 using the student ID number, they removed the student ID number from the data for the analysis.

Baseline characteristics at the start of CBME (T5), including age, sex, and RIPLS scores at each time point, were compared between the CBME 2018–2019 and 2019–2020 groups using independent-samples t-tests for continuous variables and chi-square tests for categorical variables to assess the comparability of the two groups.

Descriptive statistics were used to summarize changes over time from T1 to T4 among participants included in the primary analysis, as described below. Due to missing data at earlier time points, the number of observations varied across time points.

The primary analysis focused on changes from before CBME (T5) to after CBME (T6) and at graduation (T7), in line with the study objective of examining changes in medical students' readiness for interprofessional learning before and after CBME and whether these changes are sustained until graduation. In addition, these time points had a higher response rate and shorter measurement intervals, ensuring more robust longitudinal analysis. Earlier time points (T1–T4) were not included in the primary analysis due to substantial missing data. Participants with missing data at any of the primary analysis time points (T5–T7) were excluded, and analyses were conducted using complete cases. A one-way repeated-measures analysis of variance (ANOVA) was performed.

When the assumption of sphericity was violated, Greenhouse–Geisser corrections were applied. Effect sizes were reported as partial eta squared (ηp²).

**Figure 1:**
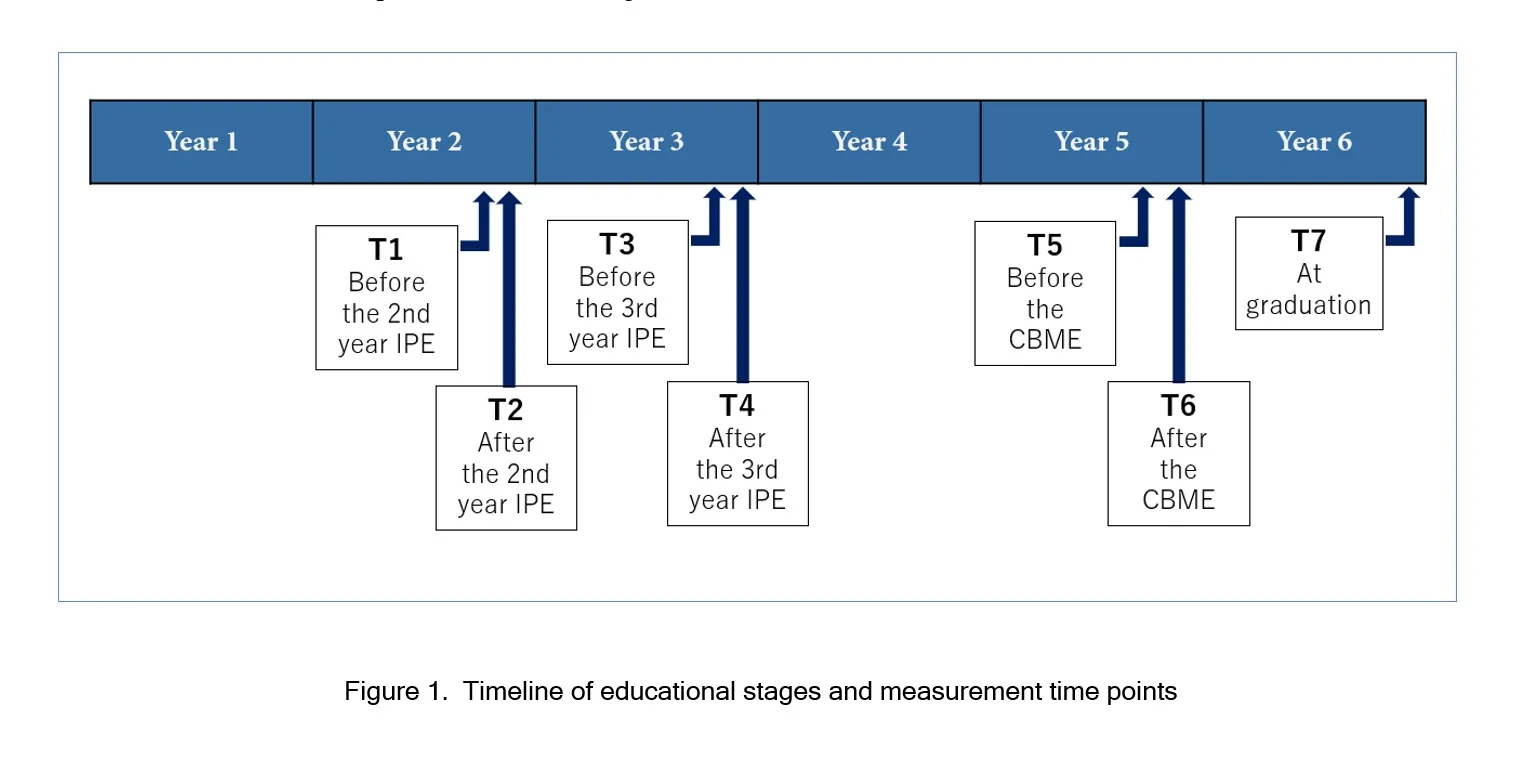
Timeline of educational stages and measurement time points

### Statistical analysis

Post hoc pairwise comparisons were conducted using Bonferroni correction, applied to all pairwise comparisons among T5–T7. Mean differences with 95% confidence intervals (CIs) were reported. A two-sided p-value < .05 was considered statistically significant. We conducted the statistical analysis using SPSS Statistics 30.0.0.0 (IBM Japan).

## Results

Participant flow is shown in Figure 2.

In the CBME 2018–2019 group, 118 students participated in CBME. One student was lost to follow-up at graduation due to repeating a year. Among the remaining students, 9 had missing data at either T5 or T6 (or both), and 4 had missing data at T7; these students were excluded from the analysis. As a result, 104 students with complete data for T5–T7 were included in the analysis.

In the CBME 2019–2020 group, 136 students were scheduled to participate in CBME; however, 35 students could not participate in the CBME from April to May 2020 owing to COVID-19–related cancellations, and one student did not provide consent to participate in the study. These students were excluded prior to analysis. One additional student was lost to follow-up at graduation due to repeating a year. Among the remaining students, 8 had missing data at either T5 or T6 (or both), and 3 had missing data at T7; these students were excluded from the analysis. As a result, 88 students with complete data for T5–T7 were included in the analysis.

In total, 192 students (104 in the CBME 2018–2019 group and 88 in the CBME 2019–2020 group) were included in the primary analysis.

Table 1 presents the baseline characteristics of participants at the start of CBME (T5). No significant differences were observed between the CBME 2018–2019 and 2019–2020 groups in age or sex.

**Table 1:** Baseline characteristics of participants at the start of CBME (T5)

Variable		CBME 2018-2019 group		CBME 2019-2020 group		p-value
Age, mean (SD), years		23.5 (1.5)		24.0 (3.4)		0.165
Gender		n	%	n	%	0.875
	Male	70	67.3	58	65.9	
	Female	31	29.8	27	30.7	
	Missing	3	2.9	3	3.4	

Given the lack of statistically significant differences between groups in baseline characteristics (age and sex) or RIPLS total scores at any time point (all independent samples t-tests were non-significant, p > .05; for example, T5: t_(__190) _= 1.85, p = .066), and the primary aim of examining overall changes associated with CBME, the two groups were combined for subsequent analyses.

Figure 3 illustrates the changes in mean RIPLS total scores across all time points. Descriptive statistics, including means, standard deviations, and sample sizes at each time point, are presented in Table 2. The mean RIPLS total score increased from T1 (73.8) to T2 (78.2), decreased at T3 (74.4), increased again at T4 (78.0), remained relatively stable at T5 (78.6), increased to its peak at T6 (82.2), and decreased at T7 (76.8). From T1 to T4, these changes represent descriptive trends. Due to missing data and academic year repetition at earlier time points, the number of observations varied across time points. Analyses for T5–T7 were based on participants with complete data (n = 192).

**Figure 2:**
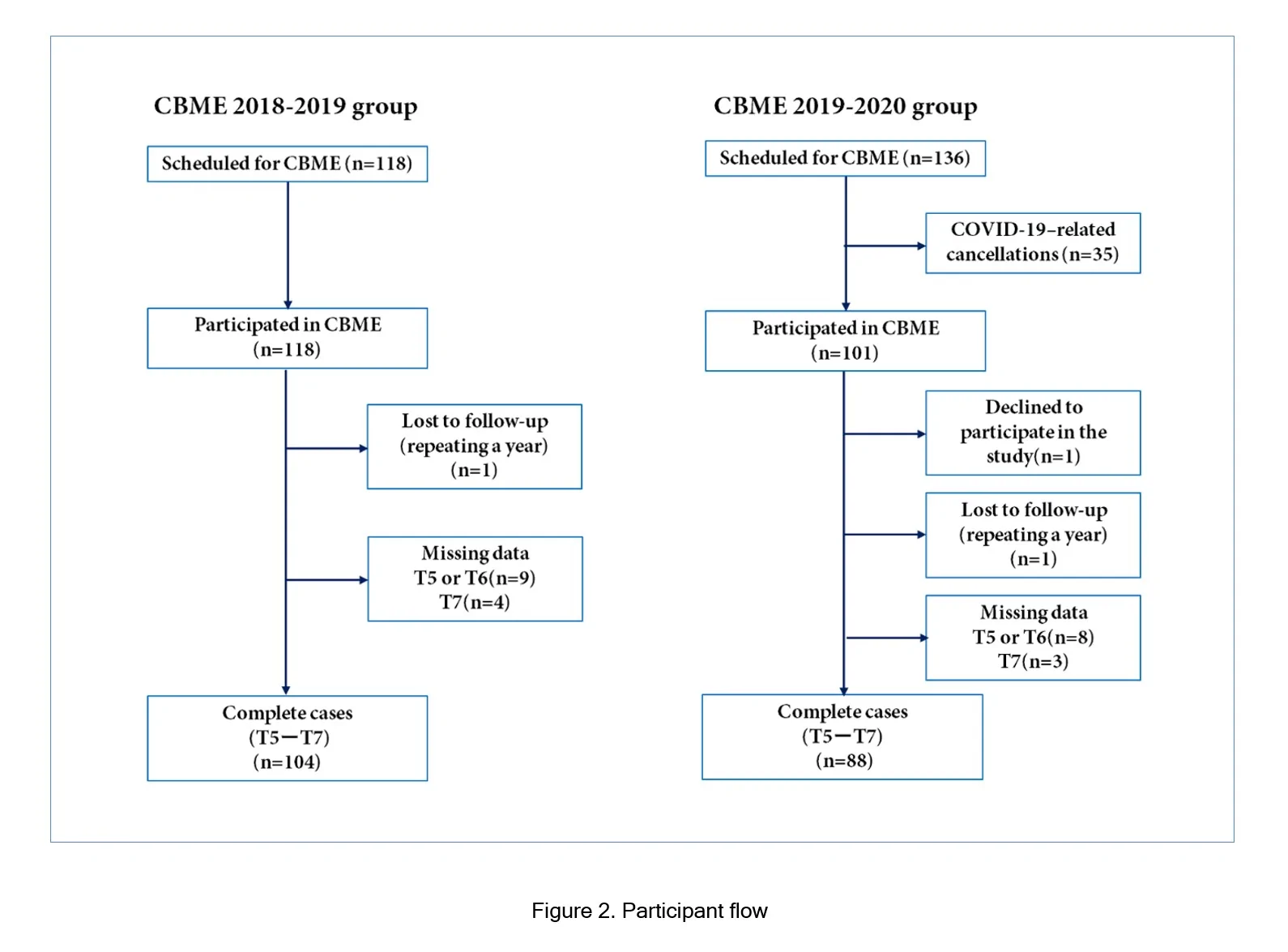
Participant flow

**Figure 3:**
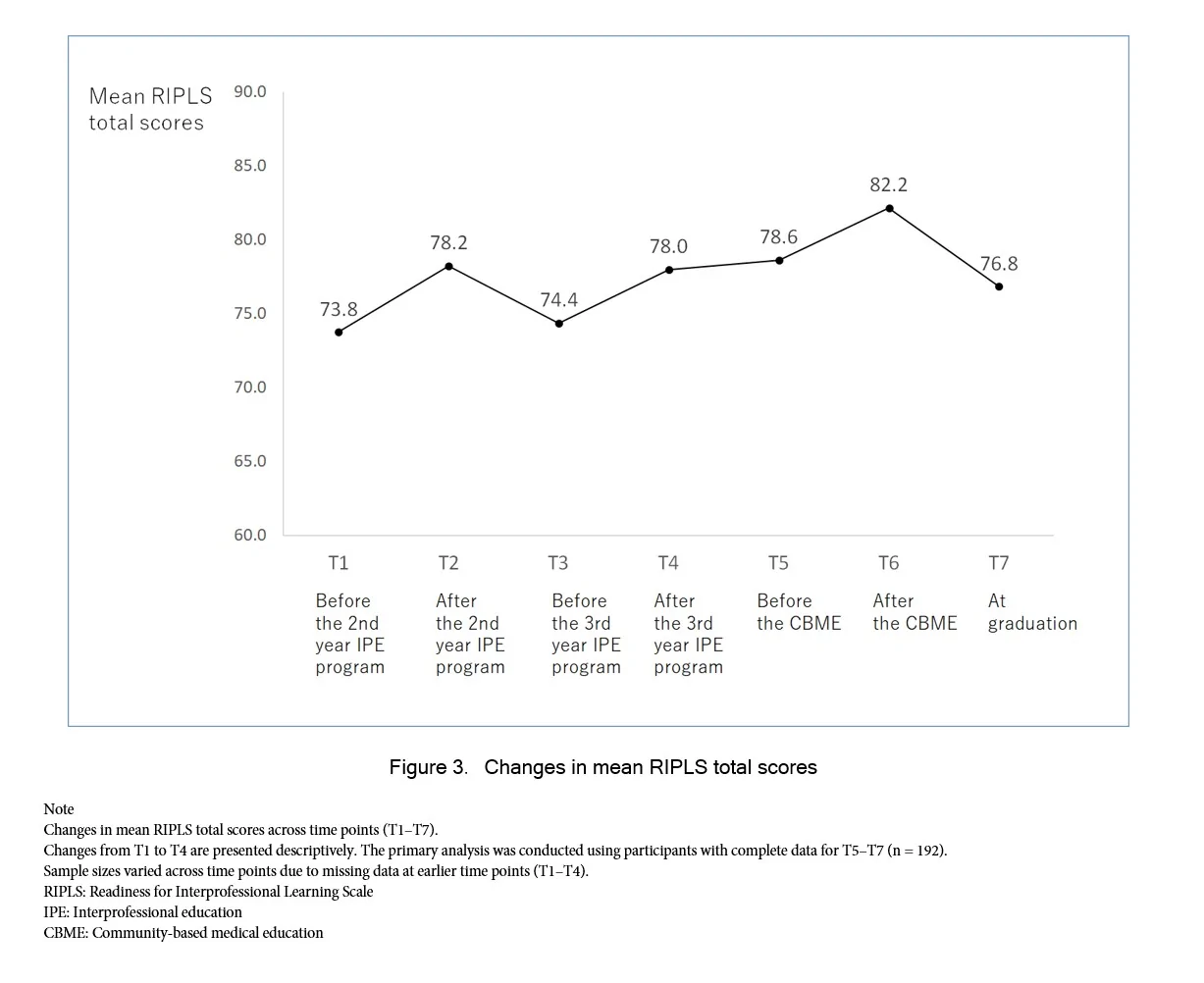
Changes in mean RIPLS total scores

The primary analysis focused on changes from T5 to T7. Mauchly’s test indicated that the assumption of sphericity was violated (p < .05); therefore, Greenhouse–Geisser corrections were applied. A one-way repeated-measures analysis of variance (ANOVA) conducted among participants with complete data for T5–T7 showed a significant effect of time on mean RIPLS scores, F (1.60, 305.28) = 31.50, p < .001, ηp² = 0.14. Post hoc comparisons with Bonferroni correction indicated that scores at T6 were significantly higher than those at T5 (mean difference = 3.51, 95% CI [2.35, 4.67], p < .001), whereas scores at T7 were significantly lower than those at T6 (mean difference = −5.31, 95% CI [−7.12, −3.49], p < .001). There was no significant difference between T5 and T7 (mean difference = −1.80, 95% CI [−3.66, 0.06], p = .062).

**Table 2:** Descriptive statistics of RIPLS total scores at each time point

Time point	n	Mean	SD
T1	168	73.8	8.6
T2	170	78.2	8.2
T3	183	74.4	8.6
T4	184	78.0	10.1
T5	192	78.6	8.8
T6	192	82.2	8.7
T7	192	76.8	11.1

RIPLS: Readiness for Interprofessional Learning Scale

SD: standard deviation

Sample sizes at T1–T4 vary across time points due to missing data and academic year repetition;

analyses for T5–T7 were based on complete cases (n = 192)

## Discussion

This study demonstrated that medical students' readiness for interprofessional learning increased after CBME and reached its peak at the completion of CBME. However, the increase in readiness following CBME was not sustained until graduation. To our knowledge, this study is among the few to longitudinally examine changes in medical students' readiness for interprofessional learning and follow students through to graduation.

The increase observed between before and after CBME suggests that CBME provides an effective learning environment for enhancing readiness for interprofessional collaboration. Clinical training offers opportunities for authentic engagement in team-based care, which may facilitate the integration of interprofessional competencies beyond classroom-based learning.

Notably, readiness for interprofessional collaboration decreases at graduation, despite reaching its peak following CBME. This finding contrasts with the findings of previous research, which suggest that students in their final year are well prepared for increased interprofessional collaboration.^15^ In Japan, the final year of medical school is often dedicated to preparing for the National Medical Practitioners Qualifying Examination, which limits opportunities for collaborative learning. This may explain the observed decrease in readiness.

Descriptive trends observed during the classroom-based IPE periods suggest short-term increases in readiness following each intervention. Previous research has shown that about half of pre-graduate IPE programs significantly increase positive attitudes toward IPE before and after the program.^5^ Similarly, this study finds an increase in medical students' readiness for IPE both before and after the classroom-based IPE. However, readiness decreases between the second and third year of the IPE program, possibly owing to a lack of opportunities to engage with other faculties or professions during this period. However, these findings should be interpreted with caution, as no formal statistical analyses were conducted for these time points due to missing data.

The revised Model Core Curriculum for Medical Education in Japan, 2022 Revision, emphasizes interprofessional collaboration as a core competency16, and includes learning objectives based on the interprofessional competency framework for collaborative practice in Japan.^17^ To ensure seamless continuity between undergraduate and postgraduate clinical training, IPE in medical school should connect to interprofessional collaboration in clinical practice. A spiral curriculum, which introduces early IPE and incorporates repeated learning at appropriate levels, is recommended in the literature.^2.^ Frequent repetition in earlier years may help sustain readiness for interprofessional collaboration, while clinical experience in later years can reinforce this readiness. Additionally, to counter the decrease in readiness observed at graduation, introducing collaboration-focused programs in the early stages of postgraduate clinical training might prove effective.

However, implementing IPE programs faces challenges, including schedule adjustments and insufficient staff numbers.^18^^,^^19^ Although an increasing number of universities inJapan are adopting IPE,^20^ many still lack continuous programs, with most offering only a single program for lower-year students.^21^^,^^22^ Online IPE, which has gained prominence since the COVID-19 pandemic, may help address these challenges by overcoming geographical and logistical barriers.^23^

Several limitations of this study warrant mention. First, because this was a single-institution study conducted in Japan, we need to generalize the results cautiously. Second, the primary analysis was conducted using complete cases, which may have introduced selection bias, as students with complete data may differ systematically from those with missing data. For example, students with complete data may have been more consistently engaged in the curriculum and progressed without interruption, which could have led to an overestimation of readiness for interprofessional learning. In addition, the CBME 2019–2020 group experienced potential disruptions related to the COVID-19 pandemic, which may have influenced learning opportunities and students' readiness for interprofessional collaboration. However, as clinical rotations were scheduled across different time periods, the impact of these disruptions may have been distributed across students, potentially limiting systematic bias. Third, students participated in interprofessional learning in small groups during classroom-based IPE and interacted with various professionals across multiple training sites during CBME. Because group composition and training environments varied across time points, potential clustering effects could not be fully accounted for in the analysis. Fourth, although no substantial differences were observed between the CBME 2018–2019 and 2019–2020 groups in baseline characteristics or total RIPLS scores, the observed changes, particularly the increase from T5 to T6, cannot be definitively attributed to the effects of CBME, as maturation or other unmeasured factors may have influenced the results. Finally, concerns have been raised about the reliability and validity of the questionnaires used to evaluate IPE,^24^ including the RIPLS instrument used in this study.^25^ Although no perfect tool exists for measuring the educational effect of interprofessional learning, the RIPLS has demonstrated internal validity in numerous studies and remains a widely used evaluation instrument for pre-graduate IPE.^26^ For consistency, we follow previous studies and use the total RIPLS score.^27^^,^^28^

## Conclusions

This study examined whether improvements in medical students' readiness for interprofessional learning following CBME are sustained until graduation. Readiness peaked after the CBME but was not maintained until graduation.

Introducing collaboration-focused training during the early stages of postgraduate clinical training and creating a seamless connection between pre-graduate IPE and postgraduate interprofessional collaboration may help sustain readiness and enhance the transition to clinical practice.

### Conflict of Interest

The authors declare that there is no conflict of interest.
